# Tincture of Benzoin in Chapped Nipples

**Published:** 1855-11

**Authors:** 


Tincture of Benzoin in Chapped Nipples.—M. Bourdel states this is the best application, whatever the extent or duration of the fissure may be. After suckling it should be freely applied with a pencil. The first application causes a burning pain for a quarter of an hour, which, however, is usually bearable, and future applications give relief instead of pain. It need not be wiped off before the infant sucks.—(*Jour. de Ch. Méd.* July. p 436.) *Ib.*

